# Meta-Analysis of Two Genome-Wide Association Studies of Bovine Paratuberculosis

**DOI:** 10.1371/journal.pone.0032578

**Published:** 2012-03-02

**Authors:** Giulietta Minozzi, John L. Williams, Alessandra Stella, Francesco Strozzi, Mario Luini, Matthew L. Settles, Jeremy F. Taylor, Robert H. Whitlock, Ricardo Zanella, Holly L. Neibergs

**Affiliations:** 1 Parco Tecnologico Padano, Lodi, Italy; 2 Istituto di Biologia e Biotecnologia Agraria, Consiglio Nazionale delle Ricerche, Lodi, Italy; 3 Istituto Zooprofilattico Sperimentale della Lombardia e dell'Emilia Romagna, Sezione di Lodi, Lodi, Italy; 4 Department of Animal Sciences, Washington State University, Pullman, Washington, United States of America; 5 Division of Animal Sciences, University of Missouri, Columbia, Missouri, United States of America; 6 School of Veterinary Medicine, University of Pennsylvania, Kennett Square, Pennsylvania, United States of America; Auburn University, United States of America

## Abstract

**Background:**

Bovine paratuberculosis (ParaTB) also known as Johne's disease, is a contagious fatal disease resulting from infection by *Mycobacterium avium* subspecies *paratuberculosis* (*MAP*). Previous studies have identified loci associated with ParaTB using different measurements to define cases and controls. The objective of this study was to combine the data from two recent studies to identify genetic loci associated with *MAP* tissue infection and humoral immune response, defined by *MAP* ELISA-positive cattle, by comparing cases and control animals for one or both measures of infection.

**Methodology/Principal Findings:**

The two populations used for the association analyses were a cohort of *MAP* tissue infected animals and control Holstein cows from the USA and the second cohort composed of ELISA-positive and ELISA-negative Holstein cows from Italy. Altogether 1190 cattle were genotyped with the Illumina BovineSNP50 BeadChip. SNP markers were removed if the minor allele frequency <0.01 or genotyping failure was >5%. Animals were removed with >5% genotyping failure. Whole genome association analyses were conducted with the GRAMMAR-CG method using two different definitions of control populations.

**Conclusion/Significance:**

The analyses identified several loci (P<5 e-05) associated with ParaTB, defined by positive ELISA and presence of bacteria in tissue compared to ELISA and tissue negative animals, on chromosomes 1, 12 and 15 and one unassigned SNP. These results confirmed associations on chromosome 12 and the unassigned SNP with ParaTB which had been found in the Italian population alone. Furthermore, several additional genomic regions were found associated with ParaTB when ELISA and tissue positive animals were compared with tissue negative samples. These loci were on chromosomes 1, 6, 7, 13, 16, 21,23 and 25 (P<5 e-05). The results clearly indicate the importance of the phenotype definition when seeking to identify markers associated with different disease responses.

## Introduction

Control of major infectious disease in livestock remains difficult, despite the detailed characterisation of the infectious agents associated with common diseases and the deciphering of their genome sequences [1]. In addition, the outcome of infection is variable among animals of the same breed or between breeds and species [2]. Consequently, host genetic control of infectious diseases is being investigated with the view that selection can be applied in livestock breeding to reduce disease incidence and improve animal health, food safety, diagnostic tools and prevent zoonoses.

Paratuberculosis (ParaTB) or Johne's disease, caused by *Mycobacterium avium* subspecies *paratuberculosis* commonly known as *MAP* in cattle, is a chronic gastroenteritis characterized by diarrhoea, weight loss, decreased milk production and ultimately death [3]. Heritability estimates for host susceptibility to ParaTB range from 0.06 to 0.10 [4], [5]. The definition of an infected animal can be based either on the presence of anti-*MAP* antibodies in the serum, or bacterial culture from tissue or faeces. Previous studies to estimate heritability were based on serology [4], [5], while recent genome wide association studies GWAS have used tissue and faecal culture of the bacterium as well as serology as the phenotypes to define infected and control individuals [6]–[11]. These different definitions of infected status may explain the different genetic loci identified as involved in the susceptibility to ParaTB. The control of diseases can be achieved using several complementary tools, one of which is selection based on molecular and epidemiological information. Studies at the molecular level can reveal how the genetic repertoire of a population controls its susceptibility to different infectious agents. Once the loci associated with susceptibility to disease are identified and understood, they can be included into breeding schemes. Outbreaks of zoonotic diseases such as porcine influenza [12], further illustrate the importance of research in infectious diseases, and the need to understand the variability among populations to such infectious agents and intra species transmission routes.

In recent years, new knowledge of cellular and molecular immune response has been obtained largely from studies using experimental models and defined genetic lines of laboratory animals. However, animal models of infectious diseases suffer from the limitation that the experimental conditions remove the sources of variation that impact the disease. In the case of livestock, there are many complex host-environment interactions which affect the presentation of diseases and their level of incidence. The use of field samples is therefore an indispensable complement to studies in animal models. However such data are often difficult to interpret, as the sources of variation are not well understood. The advances in molecular and bioinformatic tools for the analysis of genomes, together with epidemiological theory previously used only in human studies, is now being applied to studies of livestock species. Through genome-wide association studies (GWAS) using high densities of SNP markers, we are finally in a position to perform a joint analysis across datasets to increase the power and discrimination of individual studies.

Until a few years ago, it was not possible to design genome-wide scans in livestock based on field samples with unknown pedigree information as markers were not sufficiently dense to detect linkage disequilibrium between the markers and causative variations. Following the publication of the bovine genome sequence, high marker density SNP assays became available. Statistical methodology has been developed to make use of these high density marker panels to detect associations between markers and traits in populations where linkage disequilibrium extends over more limited distances. The problem now is that cattle populations generally have a high level of relatedness, especially dairy cattle where the effective population size and number of sires is very small. The hidden presence of closely related animals in the sample set results in a complex population structure and an *a priori* unequal distribution of allele frequencies between cases and controls. The resulting population stratification may inflate the rate of false positive associations between the trait and the markers, and could hide the true associations. In this study, we used a polygenic model that included the genomic kinship matrix among samples in the analyses, and multidimensional scaling to identify individual animals that had genetic backgrounds that were different from the general population of animals [13].

This article reports a joint analysis of two independent GWAS datasets to identify genes and markers associated with ParaTB susceptibility in dairy cattle. The objective was to improve the power to detect associations over what was possible in each individual study, and to investigate the consistency or heterogeneity of these associations across diverse phenotype definitions and populations. The study design was based on the joint analysis of two cohorts of animals. All animals were of the same breed and tested for the presence of antibodies for MAP or lesions in the intestinal tract. Furthermore, population stratification was assessed by MDS (multi dimensional scaling), no specific intervention was done to the animals and all samples were field samples.

## Methods

No electronic search strategy was performed to seek adequate databases to be included in the meta-analysis. The study was conducted in collaboration between two research institutions and the two databases were shared under personal agreement and communication. The databases were chosen based on the breed of the animals (Holstein-Frisian), the genotyping platform used (Bovine 50K SNP CHIP) and availability/possibility to share information. Raw genotypic datasets and phenotypic files were personally obtained from authors of the manuscript. The principal summary measure of the results of the work is a p-value associate to the single SNP tested in the combined dataset. The principal risk of bias in the study can be linked with the limited number of studies analysed, 2 cohorts, and with the different phenotypic definition that is used to define the diseases in the two datasets. No specific analysis of risk of bias was conducted on the two datasets.

### 1. Animals and case-control definition

In addition to comparing the results of GWA analysis of studies using different disease phenotype definitions, two different control definitions were also used. Group (A) controls consisted of animals that were ELISA or tissue culture negative, where a case was defined as an animal that was either positive for the *MAP* ELISA test or was *MAP* positive by culture of the ileum, ileo-caecal valve, or ileo-caecal lymph nodes. Group B controls consisted of animals that were *MAP* tissue culture negative and cases consisted of animals that were either *MAP* ELISA positive or *MAP* tissue positive.

Group A comprised 1190 animals, 590 cases (483 ELISA positive, 107 tissue positive) and 600 controls (483 ELISA negative and 117 tissue negative), prior to genotype quality checks. Group B comprised 707 animals, 590 cases (483 ELISA positive, 107 tissue positive) and 117 controls (117 tissue negative), prior to genotype quality checks.

### 1.1.2 Italian Holstein population

Samples were collected from routine ParaTB screening of Holstein cattle between September 2007 and December 2008 in the province of Lodi in Italy, in an area with a high prevalence of ParaTB. Animals were defined as ParaTB positive based on the detection of serum antibodies produced in response to *MAP* infection using the ID-screen® ELISA test (ID VET Montpellier, France). To minimize relatedness between animals, samples were selected from many herds. In total, 2818 samples from Holstein cows were collected from 119 farms, among which, 966 were chosen for the final association study; of these samples, 483 were *MAP* antibody positive (cases) and 483 *MAP* antibody negative (*MAP* negative controls). All animals were female, and cases and *MAP* negative controls were from the same farm tested on the same day. Full details of animals, DNA extraction and genotyping are given in Minozzi et al. 2010 [7].

### 1.1.3 American Holstein population

Two hundred forty-five Holstein cows from herds in New York, Pennsylvania, and Vermont were followed to culling between January 1999 and November 2007 and assessed for the presence of *MAP* in culture from both faecal and necropsy tissue samples *post mortem*. In this study, 224 samples out of 245 were used; 107 animals were classified as tissue positive for the presence of *MAP* and 117 were classified as tissue negative. Full details of animals, DNA extraction and genotyping are given in Settles et al. 2009 [10].

### 1.2 SNP Marker sets

Both sets of samples were genotyped using the Illumina (San Diego, CA) BovineSNP50 BeadChip assay, although with slightly different versions. The Italian 966 samples, plus 9 duplicated samples were genotyped using an assay with 54,001 SNPs with an average spacing of 51.5 kb and a median spacing of 37.3 kb, detailed information of markers tested can be found at the following webpage (http://www.illumina.com/products/bovine_snp50_whole-genome_genotyping_kits.ilmn). The US samples, with 1 duplicated sample, were genotyped using an assay with 55,074 SNPs with an average spacing of 49.4 kb and a median spacing of 37 kb. SNPs were mapped to the Btau4.0 assembly. The BovineSNP50 BeadChip contains 1,828 SNPs which are mapped to unassigned contigs on the Btau4.0 assembly. For both sets of samples, genotypes were called using Illumina's BeadStudio (v3.2.23) software.

### 2. Creation of a common dataset from genotypic raw data

Custom Python scripts were developed to process, store, and merge the two datasets. A unique Illumina file was created that contained 1190 samples and the common 54,001 SNPs (before quality checks) from the two datasets. All alleles were converted from BOT to TOP coding using the SNP definitions for the Illumina BovineSNP50 BeadChip as a reference and by performing a direct comparison between the genotype calls from the two datasets: this was done to ensure that the Illumina A/B genotype calls referenced identical alleles in each study. To avoid errors and potential false positives during the joint analysis, 270 SNPs were deleted because the allele assignment to the TOP strand was not reliable.

### 3. Genotype quality assurance and internal population structure analysis

Genotype quality assurance was performed within the R statistical environment using the GenABEL package implementing the “check.marker” function on the combined raw data for the two cohorts [14]. Data were quality controlled for marker call rate and minor allele frequency: markers missing >5% of the data or with minor allele frequency (MAF) <1% were removed. Samples with >5% missing data were also removed. Multi Dimensional Scaling (MDS) was used to explore population substructure and to verify the genetic homogeneity of the sample set prior to analysis. Pair-wise identities by state (IBS) were calculated for all samples based on autosomal SNPs using identity matrices implemented in the GenABEL library [14].

### 4. Genome-wide association analysis

Genome-wide association analysis was performed with the GenABEL package [14] in R using a three step GRAMMAR-GC approach, (Genome-wide association using Mixed Model and Regression - Genomic Control), with the extension of using the genomic kinship matrix estimated through genomic marker data, instead of the pedigree [13], [14]. First, an additive polygenic model was used to obtain individual environmental residuals using the polygenic function of the GenABEL library to disentangle the cryptic population structure caused by the presence of closely related animals in the sample set [13]. To account for relatedness, the variance/covariance matrix was estimated by the genomic kinship matrix, as pedigree information was not available. The relationship matrix used in the analysis was estimated using genomic data with the identity by state (IBS) “ibs” (option weight = “freq”) function of GenABEL. Second, association was tested using a simple least squares method on the residuals, corrected for cryptic relatedness, familial correlation, and independence of pedigree structure. Third, the Genomic Control (GC) approach was used to correct for the conservativeness of the GRAMMAR test, based on the estimation of the parameter lambda, which is the median of all genome-wide observed test statistics divided by the expected median of the test statistic under the null hypothesis of no association, assuming that the number of true associations is very small compared to the number of performed tests. Uncorrected p-values<5 e-07 were taken as very strong proof of genome-wide association, while p-values between 5 e-07 and 5 e-05 were considered as moderately significant associations (Wellcome Trust Case Control Consortium 2007). SNP location and gene names were based on the Btau4.0, assembly released on 4 October 2009 (http://www.ensembl.org). All analyses were carried out within the R statistical environment ((http://www.r-project.org). No review protocol exists for the meta-analysis.

## Results

### Group A

#### Results of the genotype quality assurance and internal population structure analysis

The combined dataset from both studies comprised 1190 samples and 54,001 markers. Following quality control checks, 1177 of the 54,001 markers were excluded because of low (less than 95%) call rate and 4823 markers were excluded because of low (less than 0.001) MAF. Fourteen samples were removed because of low call rate (less than 95%) and 4 were eliminated because of high autosomal heterozygosity (FDR<1%). The mean heterozygosity of the sample was 0.326, while the removed samples had heterozygosity >0.39, indicating possible sample contamination. A further 19 samples were removed due to high IBS. Mean IBS computed using genomic data with the identity by state (IBS) “ibs” (option weight = “freq”) function of GenABEL was 0.7393, based on 2000 autosomal markers, while the samples removed showed IBS higher values than 0.95. Consequently the first dataset after quality edits comprised 1153 samples and 48,001 genome-wide SNPs.

To evaluate the presence of population substructure, genome-wide SNPs that were not in linkage disequilibrium (r^2^<0.2; 13,000 SNPs) were used for the MDS plot. The population structure of the USA and Italian populations was found to be very similar as seen from the extensive overlap in the MDS plots. There were no overall differences in the genetic background of cases and controls (Figure S1). Following the removal of outliers, 1036 individuals were analysed (Figure S2).

A second quality control check was performed following the sample reduction to 1036 individuals which removed 3 SNPs because of low (less than 95%) call rate and 2715 markers because of low MAF (less than 0.01). One further sample was removed because of low call rate (less than 95%). Consequently, the final dataset comprised 1035 samples and 45,282 genome-wide SNPs.

#### Results of the analysis

**Figure 1:**
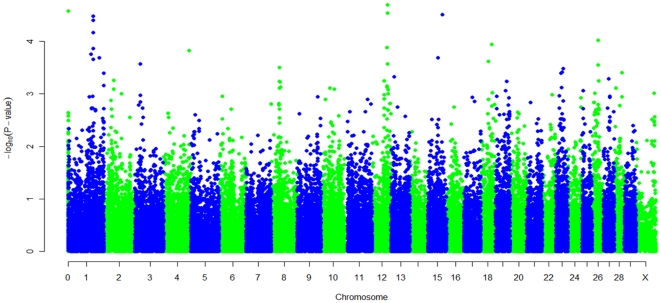
Manhattan plot displaying the −log_10_(*p*-value) results of the genome wide scan (Group A). Manhattan plot displaying the −log_10_(***p***-value) results of the genome-wide scan using the GRAMMAR-GC method with respect to their Btau4.0 genomic position for an association with *Mycobaterium avium* subspecies *paratuberculosis* defining a case as an animal that is *MAP* ELISA or tissue positive and a control as an animal that is *MAP* ELISA or tissue negative **(Group A)**.

The joint analysis of the combined data, from the two independent genome-wide studies of ParaTB, identified SNPs associated with *MAP* ELISA positive or *MAP* tissue infection positive samples, compared to controls that were either *MAP* ELISA or *MAP* tissue negative. In total, six moderately associated SNPs were identified; *ss86287819* and *rs41665666* on chromosome 12 at positions 69,663,832 and 69,599,639 (P = 2.04 e-05 and 2.66 e-05; respectively), *ss86311024* on chromosome 15 at position 66,161,046 (P = 3.07 e-05), *ss86329690* and *ss86292176* on chromosome 1 at positions 113,617,698 (P = 3.34 e-05) and 113,855,358 (P = 3.94 e-05) and *rs42743330* which is not assigned to Btau4.0 but is assigned to BTA12 at 70,812,571 on the UMD3.0 assembly (P = 2.66 e-05) (Table 1). Genome-wide “Manhattan plots” displaying the joint analysis results with respect to their genomic position, are shown in Figure 1. Evidence of population substructure was estimated by the genomic inflation factor λ = 1.12 for a basic chi-square test, and was completely corrected by the GRAMMAR-CG methodology that yielded a genomic inflation factor λ = 1.

**Table 1:** SNPs associated with positive ELISA and tissue culture test for *MAP* infection indentified by joint analysis of two genome-wide association studies in Holstein cattle.

SNP	BTA^1^	BTA Position (bp)^2^	UMD Position (bp)^3^	N^4^	effB Q.2^5^	P-value^6^
*ss86287819*	12	69,663,832	69.979.057	998	0.15	2.04 e-05
*rs42743330*	12*	-	68.866.138	998	0.17	2.66 e-05
*rs41665666*	12	69,599,639	69.620.872	1017	0.15	2.88 e-05
*ss86311024*	15	66,161,046	65.868.375	1017	−0.19	3.07 e-05
*ss86329690*	1	113,617,698	109.807.935	1017	−0.21	3.34 e-05
*ss86292176*	1	113,855,358	109.950.635	1017	−0.20	3.94 e-05

1 BTA: *Bos taurus* chromosome.

2 BTA Position: the Btau4.0 location of the SNPs on the cattle chromosome in base pairs.

3 UMD Position: the UMD3.0 location of the SNPs on the cattle chromosome in base pairs.

4 N: number of animals represented in the comparison.

5 effB Q.2.: effect of the minor allele.

6 P-values: p-values after GRAMMAR-GC test for association.

### Group B

The initial common dataset comprised 707 samples and 54,001 markers. Following the first quality control check, 1209 of the 54,001 markers were excluded because of <95% call rate and 5115 markers were excluded because of <0.001 MAF. Eight samples were removed because of <95% call rate and 2 were eliminated because of high autosomal heterozygosity (FDR<1%). The mean heterozygosity of the samples was 0.327, while the samples removed had heterozygosity values >0.41, indicating possible sample contamination. A further 7 samples were removed due to high IBS. Mean IBS computed using genomic data with the identity by state (IBS) “ibs” (option weight = “freq”) function of GenABEL was 0.7363, based on 2,000 autosomal markers, while the samples removed showed IBS values greater than 0.95. Following data cleaning, 692 samples and 47,677 genome-wide SNPs remained for the analysis.

To evaluate population substructure among the 692 animals, genome-wide SNPs that were not in linkage disequilibrium (r^2^<0.2; 13,000 SNPs) were used to produce MDS plots. The MDS plot indicated that the two populations were genetically very similar, and that there was no evidence of clustering based on the ParaTB status. After removing outliers, 619 animals remained for the analysis (Figure S3). A second round of quality control checking on these 619 animals excluded 31 of the 47,667 SNPs for call rate and 2448 SNPs for MAF. One sample was removed because of low call rate leaving 618 samples and 45,198 genome-wide SNPs for the joint analysis.

#### Results of the analysis

**Figure 2:**
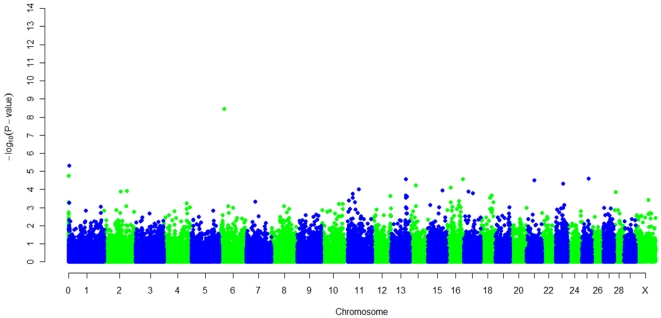
Manhattan plot displaying the −log_10_(*p*-value) results of the genome wide scan (Group B). Manhattan plot displaying the −log_10_(***p***-value) results of the genome-wide scan using the GRAMMAR-GC method with respect to their Btau4.0 genomic position for an association with *Mycobaterium avium* subspecies *paratuberculosis* defining a case as an animal that is *MAP* tissue or ELISA positive and a control as an animal that is negative to *MAP* culture in tissue **(Group B)**.

The joint analysis of the combined dataset from the two independent genome-wide studies of ParaTB infection identified several SNPs associated with *MAP*, considering samples that were either ELISA positive or *MAP* tissue positive compared to *MAP* tissue negative samples, on chromosomes 1, 6, 7, 13, 16, 21, 22 and 23, and with one unassigned SNP. Genome-wide Manhattan plots displaying the results are shown in Figure 2 and significant SNPs are provided in Table 2. Evidence of population substructure was estimated by the genomic inflation factor λ = 1.5 for a basic chi-square test, and was completely corrected by the GRAMMAR-GC methodology that yielded a genomic inflation factor of λ = 1. In total, ten loci were identified as being associated with *MAP* status; *ss86283846* on chromosome 22 (P = 1.27 e-15) at position 56,087,082, *ss86340903* on chromosome 6 (P = 3.62 e-09) at position 22,013,011, a locus on chromosome 1 by SNPs *rs29012843* and *rs29012842* at positions 3,083,368 and 3,083,498 (P = 4.82 e-06) and 7 loci with moderate evidence for an association (p-values between 1.57 e-05 and 4.65 e-05) were identified on chromosomes 7, 13, 16, 21, 23 and 25 at positions on 40,664,184, 65,977,384, 72,179,197, 3,313,513, 34,108,529, 29,929,537, respectively, and *ss86326685* an unassigned SNP on Btau4.0, but assigned to BTA26 at 2,229,651 on the UMD3.0 assembly (P = 1.74 e-05) (Table 2).

**Table 2:** SNPs associated with positive ELISA or positive tissue culture test for *MAP* infection using controls defined as samples tissue culture negative to *MAP* by joint analysis of two genome wide association studies in Holstein cattle.

SNP	BTA^1^	BTA Position (bp)^2^	UMD Position (bp)^3^	N^4^	EffB Q.2^5^	P-value^6^
*ss86283846*	22	56,087,082	53.837.358	574	−0.07	1.27 e-15
*ss86340903*	6	22,013,011	19.487.224	575	−0.07	3.62 e-09
*rs29012843*	1	3,083,368	3.591.678	600	0.03	4.82 e-06
*rs29012842*	1	3,083,498	-	600	0.03	4.82 e-06
*rs43514522*	7	40,664,184	40.766.325	549	−0.01	1.57 e-05
*ss86326685*	26^*^	-	2.091.652	589	−0.05	1.74 e-05
*ss86323177*	25	29,929,537	27.952.781	584	0.04	2.53 e-05
*ss86321275*	13	65,977,384	65.155.432	600	−0.06	2.62 e-05
*ss86317804*	16	72,179,197	73.582.939	600	−0.08	2.75 e-05
*ss86287915*	21	33,135,132	32.869.451	600	−0.13	3.15 e-05
*ss105264543*	23	34,108,529	32.251.429	596	0.03	4.64 e-05

1 BTA: *Bos taurus* chromosome.

2 BTA Position: the Btau4.0 location of the SNPs on the cattle chromosome in base pairs.

3 UMD Position: the UMD3.0 location of the SNPs on the cattle chromosome in base pairs.

4 N: number of animals represented in the comparison.

5 effB Q.2.: effect of the minor allele.

6 P-values: p-values after GRAMMAR-GC test for association.

## Discussion

Results from independent and moderately sized GWA studies rarely stand on their own and should be considered as part of a process that accumulates evidence of association [15]. In addition, complex diseases are usually polygenic in nature, where many loci with small effect are expected, and are affected by the environment. This makes it necessary to recruit a large cohort of individuals to enhance the power of the study and obtain good statistical support for the effects of the loci. One strategy is to use a joint study design to decrease the chance of false positive findings by merging studies' results or performing a combined analysis. In this study, a joint analysis of data from two cattle populations with ParaTB data was undertaken.

For livestock it is often difficult, or impossible, to increase sample size within a study either because of the difficulty in finding populations with appropriate phenotypes due to economic constrains, or because of ownership concerns that make it difficult to obtain samples from commercial populations, especially in dairy cattle. Furthermore, in order to combine studies, the overlap between marker sets needs to be maximised to optimise the accuracy of the genotyping and avoid the need for genotype imputation. Joint analysis of two or more GWA datasets of raw data is one approach to increase the evidence for the effects of genetic loci, despite the challenges associated with combining different phenotype definitions and different fixed or random effects. Even with these problems, there are many benefits to be gained as the creation of a larger sample size and the increased statistical power results in a reduced chance of false positives.

In the work presented here, we combined data from two independent GWA studies of ParaTB. The first study was performed in an Italian Holstein population. Samples were collected from routine ParaTB screening of Holstein cattle by ELISA testing. This analysis identified several regions on chromosomes 8, 9, 11, 12 and 27 (P<5 e-05) associated with disease status defined by the presence of anti-*MAP* antibodies [7]. The significant SNP on chromosome 12 fell within a previously described QTL region for ParaTB susceptibility [6] providing additional evidence that genes within these regions are involved in response to *MAP* infections. The second study was conducted in an American Holstein population and used the presence of *MAP* in both faecal and necropsy tissue as the disease phenotype. Regions on chromosomes 1, 5, 7, 8, 16, 21 and 23 were identified with moderate significance (P<1.74 e-05). Two regions, one on chromosome 3 and another on chromosome 9 were associated with the presence of *MAP* in both tissue and faeces (P<5 e-07, genome-wide Bonferroni P<0.05) [10]. Comparing the independent analyses of these two studies, a common association on Chromosome 9 was detected in both GWA studies using ELISA or *MAP* tissue infection as the phenotype.

Discrepancies between loci identified in the two previous studies [7], [10] could be the result of examining different stages of disease progression or may be linked to different diagnostic methods and hence different disease phenotype definitions. Currently, no single study has been reported that compares the genetic effects associated with different phenotypic measurements or diagnostic definitions carried out in a common set of samples for ParaTB. Consequently, the purpose of this study was to validate the SNPs identified in the previous GWA studies in a combined analysis comparing and contrasting the effects of different definitions of case and control phenotypes. Results of these analyses should help to better understand the genetic control of disease response and the potential to use the information in future breeding schemes.

The two adopted strategies of analysis (Group A and Group B) identified 6 and 11 SNPs, respectively, that were associated with disease phenotypes. The analyses were optimised as the majority of markers used in both studies were identical.

The analysis strategy based on the Group A definition where controls consisted of animals that were ELISA or tissue culture negative and a case was defined as an animal that was either positive for the *MAP* ELISA test or was *MAP* positive by culture of the ileum, ileo-caecal valve or ileo-caecal lymph nodes, identified 6 SNPs defining 3 loci associated with ParaTB on chromosomes 1, 12, and 15. Of the 6 SNPs, three were previously identified in the Italian study. The chromosome 12 QTL was identified by 3 SNPs with similar p-values and effect size [7].

Examining the region identified by the joint analysis confirmed several candidate genes located within 1 Mb of all the three markers on chromosome 12, as previously reported by Minozzi et al. 2010. Two of the significant SNPs are located within coding regions of two genes that encode a single protein, the ATP-binding cassette, sub-family C (*CFTR/MRP*), member 4 protein (*ABCC4*) which is a multi-drug resistance associated protein [16], in which polymorphisms have been shown to be involved in Crohn's disease [17].

The loci on chromosomes 1 and 15 did not reach significance in either the Italian or American studies alone. However in the American population, allele frequencies at *ss86311024* on chromosome 15 were quite different between cases (0.04) and controls (0.14) while in the Italian population the frequencies were similar (0.04 cases and 0.06 controls).

The region within 1 Mb of *ss86311024* on chromosome 15 harbours 4 genes; *LDLRAD3*, (low density lipoprotein receptor class A domain containing 3), *PAMR1* (peptidase domain containing associated with muscle regeneration 1), *CACNA1B* (calcium channel, voltage-dependent, N type, alpha 1B subunit) and *COMMD9* (COMM domain containing 9). Interestingly, both *LDLRAD3* and *CACNA1B* genes are involved in gastrointestinal diseases and polymorphisms in both these genes have been found to be associated with Crohn's disease [17] making them both strong biological and positional candidates.

Similarly, neither of the SNPs on chromosome 1 were significant in the independent studies although allele frequencies were 0.03 and 0.04 in cases and 0.06 and 0.08 in controls for *ss86329690* and *ss86292176* in the Italian and American populations. Five genes are located within 1 Mb of these SNPs, *SSRG* (signal sequence receptor, gamma), *SCL33A1* (solute carrier family 33 acetyl-CoA transporter, member 1), *LDLRAD3* (low density lipoprotein receptor class A domain containing 3), *KCNAB1* (potassium voltage-gated channel, shaker-related subfamily, beta member 1) and *GMPS* (guanine monphosphate synthetase). Polymorphisms in the gene have also been associated with Crohn's disease (p-value = 3.95025e-4) in humans [17].

The SNPs on chromosomes 1 and 15 are newly discovered associations from the joint analysis that are common to both phenotypes, i.e., antibody response and tissue burden of bacteria. These markers may be of interest for breeding schemes as they could be used to identify animals susceptible to a disease that manifests later in the productive life of the animal causing economic damage and an increased risk of contaminating other animals in the herd.

The analysis strategy based on the Group B definition compared controls (animals that were *MAP* tissue culture negative) and cases (animals that were either *MAP* ELISA positive or *MAP* tissue positive) and identified 11 loci significantly associated (P = <5 e-05) with ParaTB on chromosomes 1, 6, 7, 13, 16, 21, 22, 23, and 25 (Table 2, Figure 2). Of the 11 SNPs which appear to define 10 QTL, *rs29012843* and *rs29012842* on chromosome 1 had previously been identified in the American study using the presence of *MAP* in tissue samples as the phenotype [10]. Three genes are located within 1 Mb of these SNPs; *SOD1* (superoxide dismutase 1), *HUNK* (hormonally up-regulated Neu-associated kinase), and *SFRS15* (splicing factor, arginine/serine-rich 15). However, none of these genes seems to have biological functions linked to ParaTB, or have previously been reported to be linked to ParaTB or mycobacterial infections. Allele frequencies at these SNPs in the Italian population were similar in cases and controls indicating that the majority of the association information was related to tissue bacterial burden. None of the 7 other newly identified loci from the joint analysis reached significance in either the Italian or American studies alone.

A very strong new association (P = 1.27 e-15) was found on chromosome 22 at position 56,087,082. Interestingly, the allele frequencies at this SNP were similar between cases and controls in both cohorts. The SNP is not flanked by other SNPs associated with the disease (Figure 2), suggesting that either this is a false positive association or that the marker is in a small region of linkage disequilibrium with a causative mutation. Further information from Sanger sequencing of selected samples and analysis of the clusters generated from the Illumina bead studio, confirmed that this association is due to a bias in genotyping. Consequently, the result is not to be considered further.

None of the SNPs on chromosomes 6, 13, 16, and 21 have genes located within 1 Mb that could be considered as strong candidates for a role in ParaTB. Several of the SNPs that reached significance in the independent studies were not significant in the combined analysis, however, this does not exclude them as being associated with disease, as they may be associated with one or other of the phenotypes but not both.

In the GWA study of the Italian population with the antibody response phenotype, 6 genomic regions on BTA 8, 9, 11, 12 and 27, which were not detected in the joint analysis, were significantly associated with disease. The SNP on chromosome 9 *rs110494981* had almost equal allele frequencies between cases and controls in the combined data, whereas in the individual studies opposite frequencies were found between cases and controls in the Italian population and American populations. Therefore, it is difficult to biologically interpret this observation, as there seems to be different effects of this locus on the two phenotypes. A further possibility could be that the first finding was a false positive result.

The American study identified several SNPs linked to tissue infection on chromosomes 3, 5, 16, and 21 that were not identified in the joint analysis. The minor allele frequencies at *ss86283846* on chromosome 22 at 56,087,082 differed between the Italian (0.17) and US (0.44) populations, but in each population the frequency between cases and controls was the same, indicating a common phenotype specific trend that emerged in the joint analysis. A similar result was found for *ss86340903* on chromosome 6 at 22,013,011 and *ss105264543* on chromosome 23 at 34,108,529 bp.

In summary, combining data sets into a joint analysis of genome-wide association can improve the power for detecting and validating associations and provides the possibility of identifying new loci which were below the significance threshold in the independent studies. We combined datasets from two independent ParaTB studies. Using a genomic kinship matrix based on the Bovine SNP50 BeadChip data, we defined population relationships among samples, and show that the genetic composition of the two populations was sufficiently similar to undertake the joint analysis. In performing this analysis, using two distinct phenotypic descriptions of disease, we were able to confirm loci identified in the independent studies, and to identify new loci through increased power, presumably where the biology underlying the two phenotypes coincided. However, other loci found in the original studies were lost, again presumably where different mechanisms underlie the two phenotypes. The limitation of the study could be the phenotype definition used in the two studies, that has limited the possibility of confirming all previous results, but has at the same time enabled to find markers that are common to both diagnostic measures. Further work may be carried out by adding data from other independent GWA studies to this analysis. The concept applied here, of using datasets from two studies using the same and different trait measures may help to decipher the genetic architecture of complex infectious polygenic disease traits which require very large sample sizes to have sufficient power to detect risk loci with sufficient statistical support.

In conclusion, the results of the joint analysis of two single GWA studies confirmed previous findings and identified new genomic regions and candidate genes involved with specific and general immune response to ParaTB and have increased the overall understanding of the genetics of paratuberculosis and can be of great advantage in increasing the knowledge for genome based selection in livestock.

## Supporting Information

Figure S1 **Multi Dimensional Scale plot of the entire sample set of animals belonging to both the American and Italian population indicating cases in blue and controls in red.** Cases and controls are defined as follows (case = positive to Tissue or Elisa for MAP, control = negative to Tissue or Elisa for MAP).
(BMP)

Figure S2 **Multi Dimensional Scale plot of the entire sample set of animals belonging to both the American (blue) and Italian population (red) indicating with the red circle the cluster of animals included in the association analysis.**
(TIF)

Figure S3 **Multi Dimensional Scale plot of the sample set of animals belonging to both the American and Italian population effectively used for the association analysis after outlier removal indicating cases in blue and controls in red.** Cases and controls are defined as follows (case = positive to Tissue or Elisa for MAP, control = negative to Tissue for MAP).
(TIFF)
