# Supplementary figures and images for: Meta-Analysis of Two Genome-Wide Association Studies of Bovine Paratuberculosis
Source: PLoS One. 2012 Mar 2;7(3):e32578. doi: 10.1371/journal.pone.0032578 (PMC3292576; doi:10.1371/journal.pone.0032578)

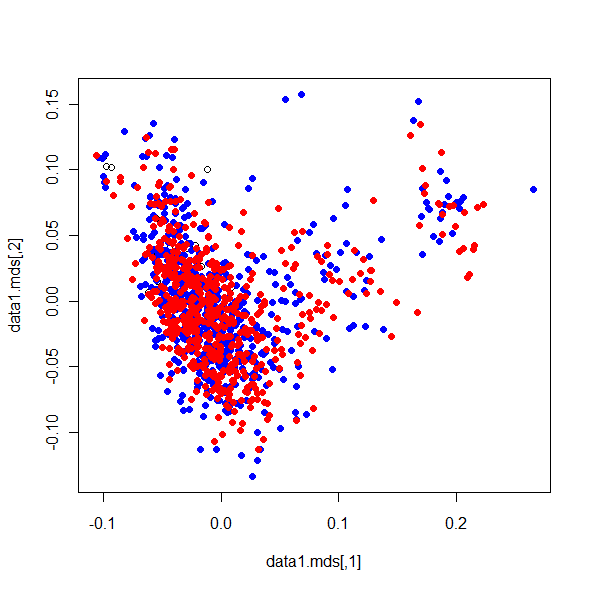

Supplement: Figure S1 — Multi Dimensional Scale plot of the entire sample set of animals belonging to both the American and Italian population indicating cases in blue and controls in red. Cases and controls are defined as follows (case = positive to Tissue or Elisa for MAP, control = negative to Tissue or Elisa for MAP). (BMP) [file pone.0032578.s001.bmp]

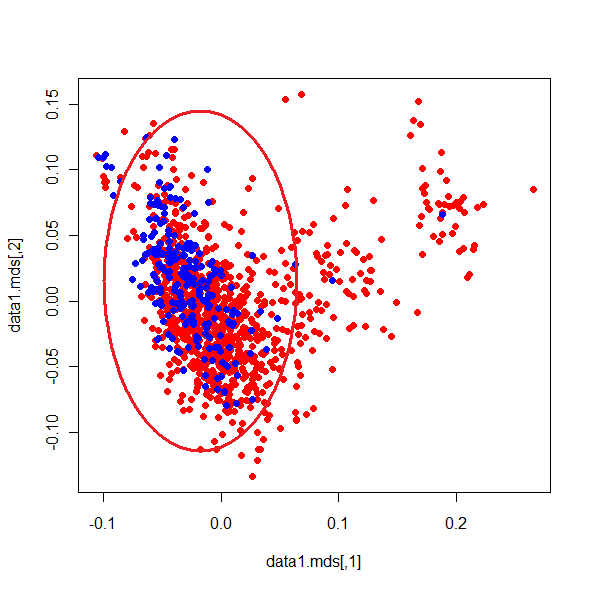

Supplement: Figure S2 — Multi Dimensional Scale plot of the entire sample set of animals belonging to both the American (blue) and Italian population (red) indicating with the red circle the cluster of animals included in the association analysis. (TIF) [file pone.0032578.s002.tif]

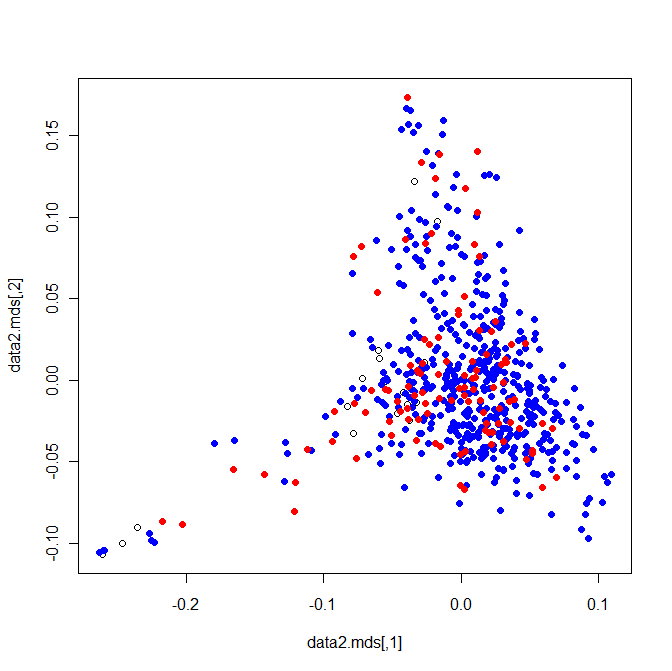

Supplement: Figure S3 — Multi Dimensional Scale plot of the sample set of animals belonging to both the American and Italian population effectively used for the association analysis after outlier removal indicating cases in blue and controls in red. Cases and controls are defined as follows (case = positive to Tissue or Elisa for MAP, control = negative to Tissue for MAP). (TIFF) [file pone.0032578.s003.tiff]
